# Conscious Self-Regulation as a Meta-Resource of Academic Achievement and Psychological Well-Being of Young Adolescents

**DOI:** 10.11621/pir.2023.0312

**Published:** 2023-09-30

**Authors:** Varvara I. Morosanova, Tatiana G. Fomina, Irina N. Bondarenko

**Affiliations:** a Psychological Institute of the Russian Academy of Education, Moscow, Russia

**Keywords:** conscious self-regulation, psychological well-being, resource approach, meta-resource, longitudinal study, adolescence, transition from primary to secondary school

## Abstract

**Background:**

The role of conscious self-regulation in determining students’ psychological well-being and academic performance is considered in the context of the fundamental problem of the regularities of young adolescents’ development.

**Objective:**

To reveal the role of meta-resources of conscious self-regulation in determining young adolescents’ psychological well-being and academic performance.

**Design:**

Sample: 500 students in 4th- to 6th grade (10–12) in general schools, 149 of whom participated in a three-year longitudinal study. The Self-Regulation Profile of Learning Activity and the Well-Being Manifestation scales were used.

**Results:**

Conscious self-regulation and academic performance increase significantly in fifth grade and decrease in sixth grade. On the contrary, psychological well-being is characterized by a systemic positive dynamic. A typological analysis identified the levels of psychological well-being of students growing, stable, and declining during the transition period. It was found that the general level of conscious self-regulation made a significant positive contribution and is a universal resource for students’ psychological well-being and academic performance. Special regulatory resources for academic performance are described, depending on the trajectory of changes in psychological well-being. Increased well-being is determined by the regulatory competencies of Planning and Evaluation of results and its stability by Planning, Modelling, Flexibility, and Responsibility. The general level of self-regulation, regulatory competencies, Planning, Programming and Responsibility mediate in the relationship between student psychological well-being and academic performance. A longitudinal study found that self-regulation has a long-term positive effect on student psychological well-being and academic performance.

**Conclusion:**

Conscious self-regulation is a meta-resource that makes a significant contribution to both the psychological well-being and academic performance. Mediator and prognostic effects of self-regulation on these properties create a psychological basis for practical work.

## Introduction

The scientific problem of the regularities of psychological development of young adolescents in grades 4–6 (ages 10–12) at a general education school has recently attracted the attention of researchers (Fomina & Morosanova, 2019; Ng, Huebner, & Hills, 2015; van Genugten, Dusseldorp, Massey, & van Empelen, 2017). This period covers the transition from primary to secondary school, takes three years, and is accompanied by a number of quantitative and qualitative changes in the adolescents’ psyche. The questions of what psychological resources are necessary for solving age-related tasks of realizing one’s own behavior, understanding one’s individual characteristics, mastering the ability to build relationships, and developing social activity continue to be relevant. Identification of these resources to maintain psychological well-being and academic performance under changing learning conditions in transitional periods remains a significant and not yet solved task.

In spite of the fact that there has been considerable research on the cognitive and personal development of young adolescents, theoretical studies of age-related changes in the regulatory sphere are fragmentary and available empirical data are often contradictory. And while the personal (Potanina & Morosanova, 2022) and motivational (Gordeeva, Sychov, & Stepanova, 2022) spheres in relation to school success and psychological well-being have been fairly well studied (Gordeeva et al., 2022; Tikhomirova et al., 2020; Veraksa et al., 2022), the contribution of conscious self-regulation (Morosanova & Fomina, 2016) to achieving academic success without sacrificing psychological well-being has been poorly studied and is the subject of the present article.

### Conscious Self-Regulation in Achieving Educational Goals

The relevance of studying conscious self-regulation in the field of education has been often emphasized by Russian and foreign researchers (Gestsdottir et al., 2017; Morosanova, 2021; Veraksa et al., 2021). Regulatory resources are the psychological basis for the formation of universal learning competences and the achievement of meta-outcomes of education (agentive, regulatory, and personal), which are indicated in the Russian State Educational Standards (FSES OOO) as the goals to be achieved at all the levels of study. Data on the age-related characteristics of conscious self-regulation are in demand not only in psychological science, but also in pedagogical support for the achievement of educational meta-results.

### A Resource Approach to the Study of Conscious Self-Regulation (SR)

The position of adolescents as subjects of education at a new level of studies raises the issue of developing their conscious self-regulation in order to set new goals, successfully achieve them, and maintain an optimal level of psychological well-being. The present study was carried out on the basis of a resource approach to conscious self-regulation (Morosanova, 2016, 2017, 2021). We consider conscious SR to be the control level of an integral system of human psychic self-regulation. This level is realized by a person’s universal and special regulatory competencies, allowing him or her to consciously and independently set goals and manage their achievement. Our empirical studies of recent years have shown that these competencies make a direct contribution to successful activities and also serve as a psychological means for mobilizing, integrating, and mediating the influence on a person’s behavior of various subsystems of cognitive, personal, and psychophysiological resources and human reserves. In this sense, conscious self-regulation is a controlling meta-resource for successful solution of a person’s life tasks.

This meta-resource is implemented by two groups of regulatory competencies. The cognitive ones are consistently manifested in various types of activity and provide for planning one’s goals, modeling significant conditions for their achievement, programming, evaluating results, and correcting actions. Intrapersonal competencies are the ways of regulating behavior and relations with the outside world (flexibility, reliability, independence, initiative, etc.). The regulatory meta-resource has a hierarchical structure and is implemented through universal and special resources, which differ according to the scale of the tasks being addressed.

The heuristic value of the resource approach to the study of conscious SR is demonstrated by results obtained in the fields of professional activities (Kondratyuk & Morosanova, 2022; Morosanova et al., 2020b), psychological health and stress resistance (Morosanova et al., 2021b), and educational activities (Morosanova & Fomina, 2016). Surveys of schoolchildren have made it possible to empirically verify the theoretically substantiated idea that conscious self-regulation is a meta-resource for achieving educational goals, which contributes to the productive aspects of achieving these goals and serves as a mechanism for coordinating, mediating, and accumulating the entire palette of individual psychological properties used by students to solve various problems, including learning, self-education, and professional self-determination. Thus, on a sample of students in grades 7–11, it was shown that the general level of SR, along with non-verbal intelligence, acts as a universal resource for academic achievement (Morosanova & Fomina, 2016) and project activities (Morosanova & Filippova, 2021). The same is true for high achievement on the Unified State Exams (USE), in combination with SR reliability as a special resource for academic success under stressful testing conditions (Morosanova & Filippova, 2019). The regulatory competence of modeling significant conditions turned out to be a special resource for success in mathematics at school (Fomina & Morosanova, 2019). For the first time, similar patterns were replicated for success in learning one’s native language (Russian) (Morosanova et al., 2021a). An important result in terms of maintaining students’ health and well-being was obtained in relation to SR and school anxiety: regulatory competencies act as mediators reducing the risk of developing high levels of test anxiety (Morosanova & Fomina, 2017).

### Psychological Well-Being (PWB) of Young Adolescents

Studies in the field of educational psychology show that the academic performance of students with a high level of PWB is significantly higher than that of their peers with low PWB. The same is true for school engagement, self-efficacy, social adaptation, and test anxiety level (Bondarenko et al., 2020; Fomina et al., 2021; Morosanova et al., 2020a). We define psychological well-being as a construct that ensures the functional state of a student and includes a number of components. Some of them are associated with experience of satisfaction, and others with realization of the needs, meanings, and goals of the personality (Morosanova et al., 2018).

During the transitions from 4th to 5th grade and from 5th to 6th grade, the academic performance and PWB of some students stays at a fairly high level and does not undergo significant changes, while other students demonstrate an increase or decrease of these characteristics. A decline in well-being cannot but cause concern. Studies have shown the significance of life satisfaction among adolescents for the general trajectories of their age-related development and positive functioning in the future (Blakemore & Mills, 2014; Calmeiro et al., 2018; Caprara et al., 2017; Orben, et al., 2020). In this regard, longitudinal studies are of particular scientific importance, as they permit more substantiated conclusions about the factors influencing PWB dynamics. It has been repeatedly demonstrated that conscious self-regulation is one such factor (Morosanova et al., 2018; Pandey et al., 2018). In particular, regulatory competences mediate the risk of developing a high level of assessment anxiety (Martin & Steinbeck, (2017). According to a meta-analysis, this can predict a wide range of significant manifestations of PWB in children and adolescents, such as the quality of interpersonal interaction and mental health (Robson, Allen, & Howard, 2020). Moreover, more and more researchers emphasize the importance of including practical methods for developing self-regulation in programs for improving the PWB of adolescents in order to increase their satisfaction with life (van Genugten et al., 2017).

### The Relationship Among Self-Regulation, Psychological Well-Being, and Academic Achievement

The relationship among PWB, SR, and academic performance during the transition from elementary to secondary school is changing. It has been shown that young adolescents are characterized by a drop in academic motivation, which inevitably leads to a decrease in academic achievement, and this negatively affects their subjective well-being (Martin & Steinbeck, 2017). In parallel, a weakening is observed in the relationship between academic achievement and students’ PWB (Yang et al., 2019). In contrast to these trends, SR is a reliable predictor of both academic achievement and well-being of adolescents, which argues in favor of understanding SR as a meta-resource for solving various life problems, such as overcoming academic failure and academic stress, and achieving professional self-determination, etc. (Fomina & Morosanova, 2019; Gestsdottir & Lerner, 2008; Morosanova, 2017, 2022).

The age period under study has not been investigated sufficiently due to the weak ability of young adolescents to reflect and the non-linear nature of the ongoing psychological processes. The great variability of individual characteristics in the development and formation of the studied parameters led to designing various strategies for organizing empirical research and data processing to solve the problem.

### Current Study

The main hypothesis of this study is that self-regulation, as the controlling meta-level of the system of universal and special regulatory resources for achieving educational goals: directly affects academic success, and mediates the influence of other individual reserves on it, in particular, that of psychological well-being.

The study’s purpose is to reveal the meta-resource role of conscious self-regulation in determining the psychological well-being and academic performance of young adolescents.

To achieve this goal, the following research tasks were set:

1. to determine the dynamics of conscious self-regulation, psychological well-being, and academic achievement of students based on the data of a three-year longitudinal study;
2. to identify universal and special regulatory resources of academic achievement for students with different dynamics of PWB;
3. to assess the mediator effects of conscious SR (as well as of certain regulatory competencies) in the relationship between the students’ psychological well-being and academic achievement;
4. to substantiate the long-term effects of self-regulation on the psychological well-being and academic performance of students.

## Methodology

### Participants

The sample was formed from 4th- to 6th-grade students of Russian general education schools in Moscow and Kaluga. More than 500 schoolchildren took part in the study at its various stages. The sample of fourth graders consisted of 402 people (53% boys, mean age 10.20, standard deviation 0.50). The sample of students involved in the study in both the 4th and 5th grades was 239 people (48% boys). The sample of 6th-grade students included 185 people (50% boys, mean age 11.95, standard deviation 0.50). The total sample of the longitudinal study from grades 4–6 (children who took part in three measurements) was 149 people (50% boys).

### Methods

The main research instruments included “Self-Regulation Profile of Learning Activity Questionnaire, SRPLAQ-M” (Morosanova & Bondarenko, 2017) and “Well-Being Manifestations Measure Scale” (Morosanova et al., 2018). As an additional instrument, the study used “Big Five - Children’s Version”, adapted in Russian (Malykh et al., 2015). We also collected data on academic achievement across all age groups and calculated the average score of the final grades in the main academic subjects.

### Procedure

Depending on the problems being solved, these studies implemented either a cross-sectional or a longitudinal design. The cross-sectional data were used to identify a significant relationship among conscious SR, PWB, and academic achievement (including mediating effects), while the longitudinal data were used to reveal their dynamics and prognostic effects. For statistical data analysis, the study used IBM SPSS Statistics, version 26: primary statistics (mean values, standard deviations, etc.), correlation analysis, cluster analysis, and Wilcoxon test. The resource nature of conscious SR was studied by means of the Stepwise Regression method, which allowed us to assess the SR contribution to academic achievement and PWB. An integrative indicator of SR development (general SR level) was used to test the assumption of the universality of the regulatory resources for achieving educational goals, and particular cognitive and personal regulatory indicators were used to assess the contribution of special resources. Cross-longitudinal structural modeling was performed by means of the AMOS 23 program. To reveal the mediating role of self-regulation, mediator analysis was used in accordance with the classical scheme, as well as the Sobel test.

## Results

Dynamics of Psychological Well-Being, Conscious Self-Regulation, and Academic Achievement: Individual Typological Trajectories

**Figure 1.**
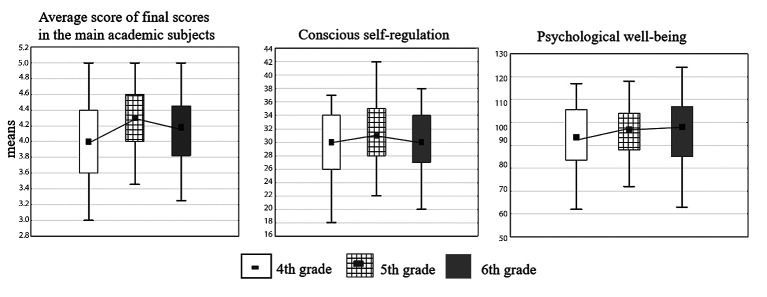
Dynamics of students’ PWB, academic achievement, and self-regulation during their transition from primary to secondary school (three longitudinal points), means and variances

*Note. Conscious self-regulation — integrative indicator calculated as sum of values for all SR features; Psychological well-being — integrative indicator calculated as sum of values for all PWB components*

The first research task was aimed at identifying the dynamics of conscious self-regulation, psychological well-being, and academic performance of the students. A longitudinal study of students from grades 4 to 6 made it possible to establish a number of regularities. *Figure 1* shows averaged data characterizing the revealed dynamics.

In the period under study, the students’ PWB is gradually increasing, while a significant improvement in academic performance and SR, which occurs during the transition from primary to secondary school, is replaced from the 5th to the 6th grade by a slight deterioration. The dynamics, therefore, show that the contradiction between activity that develops the cognitive sphere (learning), and activity aimed at understanding the relationship between people (communication), is resolved in 6th grade in favor of communication, which is manifested in a decrease in academic achievement. Indeed, according to D.B. Elkonin’s age periodization of psychic development, the outstripping development of the intellectual-cognitive sphere of the psyche in this period is replaced by development of the need-motivational sphere. Accordingly, such neoplasms as deliberateness, an internal plan of action, self-control, and reflection give way to the desire for “adulthood”, the formation of self-esteem, and submission to the norms of social life.

The SR dynamics are similar to those for achievement. Here it is important to keep in mind that, being a self-assessment indicator, conscious SR may reflect another trend of the period under study. The end of 5th grade corresponds to the transition from the first stage of adolescent development (10–11 years) to the second one (12–13 years). If at the first stage adolescents “accept” themselves, being able to identify negative traits, then at the second stage they are characterized by situationally negative self-esteem, which can be expressed in a decrease in their SR subjective assessment in 6th grade.

The rise in academic achievement, PWB, and SR during the transition from 4th to 5th grade undoubtedly requires a more careful and detailed investigation of the psychological resources providing this improvement. Particularly interesting in this regard is the continuous growth of PWB despite the crises of this age.

The rise in PWB does not negate the fact that, moving from 4th to 5th grade, some students simply maintain the previously achieved level of PWB, while others do not have enough resources to do so.

Therefore, we tested the assumption that the trajectories of PWB and academic performance are determined, first of all, by the regulatory resource. Since academic performance is also determined by personal and motivational features, these were also taken into account when building regression models.

### Universal and Special Regulatory Resources of Students with Different Trajectories of Change in Psychological Well-Being and Academic Achievement

To solve the second research task, we used regression analysis. Studies show that general SR level is a universal resource for PWB, regardless of the PWB trajectory, whereas special regulatory resources for PWB require more detailed consideration. To this end, the sample was divided into groups with different PWB dynamics: decreasing, stable, and increasing PWB.

With regard to the special regulatory resources of PWB, the following data were obtained. The increase in PWB is determined by such SR features as Planning and Result Evaluation; the stability of PWB by Planning, Modeling, Flexibility, and Responsibility. The PWB resource in the group with reduced well-being is Result Evaluation. It is worth noting the importance of objective feedback on the results achieved by the student, regardless of group affiliation. This could be the assessment of learning activities by adults as an opportunity to earn respect of the teacher and parents, or comparison with classmates’ results, which allows one to take a worthy place among peers, as well as the self-esteem which starts to form.

With respect to specific regulatory resources for academic achievement, the contribution of self-regulation was assessed for the groups with different well-being dynamics.

We confirmed the assumption that students with a decrease in PWB show a negative tendency in academic performance when they move to secondary school.

The identified regulatory predictors of academic achievement in the groups with different PWB dynamics make it possible to assess the regulatory resources allowing students to overcome the difficulties of the transition period. Numerous studies of the SR contribution to the achievement of learning goals demonstrate that it varies from 20 to 35% in different samples. In this case, we can talk about a universal regulatory resource. When evaluating the SR contribution to the achievement of specific learning goals, it is preferable to identify regulatory cognitive and intrapersonal features that make up the student’s special resources in relation to these goals (*Table 1*).

**Table 1:** Specific regulatory resources of academic achievement during transition from primary to secondary school in groups with different dynamics of psychological well-being

4th grade			5th grade			6th grade		
	Beta	*p-*level		Beta	*p-*level		Beta	*p-*level
**Decreased PWB**								
R2 = 0.32, F(8, 20) = 2.61, *p* < 0.03			R2 = 0.29, F(4, 103) = 11.83, *p*<0.00			R2 = 0.46, F(10, 28) = 4.19, *p*<0.001		
Planning	0.65	0.01	Modeling	0.86	0.04	Achievement motivation	0.64	0.00
Modeling	0.45	0.03	Responsibility	–0.60	0.01	External motivation	–0.62	0.00
			Anxiety	0.86	0.01	Cognitive motivation	0.38	0.03
			Achievement motivation	0.69	0.02	Neuroticism	0.33	0.02
						Flexibility	0.27	0.05
**Stable PWB**								
R2 = 0.31, F(6, 17) = 13.83, *p* < 0.00			R2 = 0.27, F (9, 115) = 6.22, *p* < 0.00			R2 = 0.59, F(13, 46) = 7.68, *p* <0.000		
Planning	0.31	0.00	Planning	0.21	0.04	Openness	0.69	0.00
Flexibility	–0.18	0.01	Achievement motivation	0.42	0.00	Parents’ motivation Respect	–0,27	0.01
			Anxiety	–0.26	0.01	Planning	0.25	0.03
			Openness	0.62	0.00	Conciseness	–0.58	0.01
						Achievement motivation	0.28	0.02
						Anger	–0.27	0.03
						Modeling	0.21	0.07
**Increased PWB**								
R2 = 0.47, F (6, 19) = 4.65, *p* < 0.00			R2 = 0.79, F (10. 11) = 8.87, *p* < 0.00			R2 = 0.63, F (11. 32) = 7.69, *p* <0.00		
Programming	0.53	0.01	Planning	0.49	0.01	Openness	0.60	0.00
Modeling	0.50	0.01	Responsibility	0.64	0.00	Flexibility	0.34	0.00
Neuroticism	0.50	0.01	Independence	0.31	0.02	Programming	–0.38	0.01
Openness	0.27	0.00	Anger	–0.82	0.00	Parents’ motivation Respect	0.26	0.01
			Extraversion	–1.64	0.00	Extraversion	–0.47	0.05
			Agreeableness	1.83	0.00	Responsibility	0.35	0.01
						Anxiety	–0.36	0.00

Table 1 shows how the special regulatory resources of academic achievement change in the groups with different PWB dynamics at the stage of transition from primary to secondary school, following a change in the character of learning tasks. The table shows that by 6th grade, the system of predictors of academic achievement becomes more complicated. While in 4th grade it is self-regulation and personality features, in the 5th grade achievement motivation and anxiety are added, and in 6th grade it is a complex system of regulatory, motivational, personal, and emotional properties.

Thus, in 4th grade, for the group with a decrease in PWB, such resources are the competencies of Planning and Modeling, which indicates the efforts consciously made by students to achieve better results. Progress in the group with stable PWB is determined by the level of Planning and Regulatory Flexibility (with the negative sign), as well as the pronounced personal disposition Openness to new experience. The positive contributions of Planning and Openness indicate that not only volitional processes of self-organization have a positive effect on the progress of school-children; equally important here is their interest in learning new things, the desire to expand their knowledge in the subjects of the curriculum. Reduced Flexibility is likely to keep the student’s attention on the discipline being studied. Progress in all significant subjects in the group with an increase in PWB is also determined by self-regulation and personal dispositions. This group included students who are able to build and organize stable, step-by-step progress towards a goal, using all the factors contributing to its achievement (the positive contribution of Programming and Modeling).

Presenting the analysis of resources and predictors of academic performance in 5th grade, we will focus only on the regulatory competencies. In the group with a decrease in PWB, the main resource for academic success is the students’ ability to identify the factors significant for achieving a goal (Modeling). The decrease in regulatory Flexibility and Responsibility leads to a decrease in academic achievement. In the stable PWB group, academic success is determined by the ability to set learning goals (Planning). In the group with an increase in PWB, Responsibility, Planning, and Independence make a positive contribution to the students’ performance.

Summarizing the results, we note that a distinctive feature of students with high PWB and high academic achievement is the regulatory property of Responsibility, reduced Extraversion, and a high level of positive emotions in relation to both the learning process and its participants (high Agreeableness). It is important to emphasize that the fundamental difference between a universal resource and a special one is the greater connection of the latter with a differential psychological basis (cognitive and personal) of the individual, as well as the regulatory skills accumulated in the individual’s experience of achieving various goals. It is this regularity that the resulting equations have demonstrated. A special regulatory resource corresponds only to a certain class of goals or even to one specific task. Its content and degree of development bears the imprint of specific actions necessary to achieve a particular goal.

In 6th grade, along with a general decline in both self-regulation and academic performance (*Figure 1*), we note that in the group with negative PWB dynamics the only special regulatory resource is Flexibility. Obviously, the failure to set learning goals and to take steps to achieve them, the refusal to be responsible for the results, are the cause of both reduced well-being and reduced academic performance, compared to 5th grade. In the stable PWB group, performance is determined primarily by Openness to new experience and regulatory competencies in Planning and Modeling. The only thing that does not allow the students of this group to achieve high results is their reduced conscientiousness. And, finally, students in the group with growing PWB achieve high performance through their high responsibility and flexibility. We note the negative contribution of Programming to academic performance, which may not be a general pattern and requires further research.

### The Mediating Role of Regulatory Resources in the Relationship Between Psychological Well-Being and Academic Achievement of Young Adolescents

The research task of assessing the mediating effects of conscious SR in the relationship between psychological well-being and academic achievement was addressed by means of mediator analysis. The basis for setting this task was the obtained results on the significant relationship of the level of SR development with both PWB and academic performance.

To test the assumption about the mediating contribution of various regulatory competencies to the relationship between psychological well-being and academic performance in young adolescents, a mediator analysis was performed in accordance with the classical scheme and the Sobel test. We relied on a confirmed reciprocal relationship between the academic achievements of the adolescents and their well-being (not only psychological, but also its other form — subjective well-being). However, we took into account that the direction of the causal relationship between PWB and academic performance in different periods of study remains debatable.

**Figure 2.**
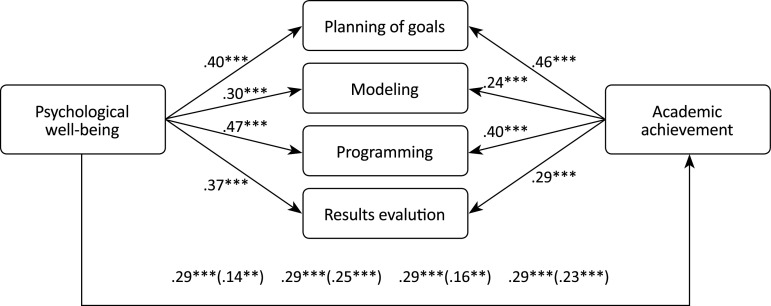
Effect of psychological well-being on academic achievement, mediated by specific regulatory resources

Note. Standardized regression coefficients and direct effect coefficients are shown. The total indirect effect coefficient of psychological well-being on academic achievement is shown in parentheses. **p < .01, ***p < .001.

**Figure 3.**
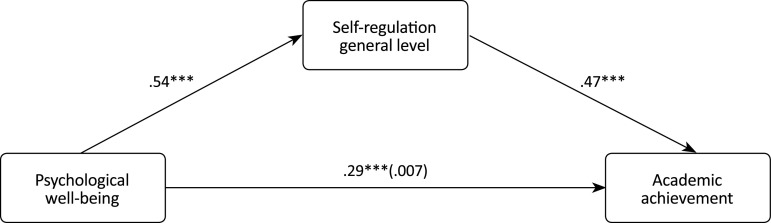
Effect of psychological well-being on academic achievement, mediated by general level of self-regulation (universal resource)

Note. Standardized regression coefficients and direct effect coefficients are shown. The total indirect effect coefficient of psychological well-being on academic achievement is shown in parentheses. ***p < .001.

To answer the question of whether self-regulation (as well as certain regulatory competencies) is a mediator of the relationship between PWB and student achievement, we used a statistical analysis of mediation as one of the forms of structural modeling. To test the research hypothesis, we analyzed several mediator models, which successively included individual cognitive regulatory competencies as mediators (*Figure 2*), as well as an integral indicator of the general SR level (*Figure 3*). The dependent variable was academic achievement; the independent variable was students’ PWB.

The most significant and high mediator effect was found for the Planning and Programming competencies (*Figure 2*). The highest mediator effect was demonstrated by the general SR level. Thus, the study confirmed the hypothesis that the development of conscious self-regulation is a universal resource for psychological well-being and academic achievement, which can affect academic performance both directly and through the relationship with special regulatory resources for the PWB of students.

### Predictive Effects of Conscious SR Influence on Academic Success and PWB of Students During Their Transition from 4th to 5th and 6th Grades

**Figure 4.**
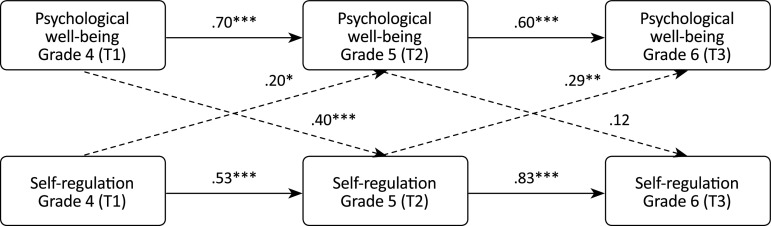
A three-wave cross-lagged panel model of Self-Regulation and Psychological Well-Being

Note. Standardized coefficients are presented. ***p < .001, **p < .01, *p < .05

**Figure 5.**
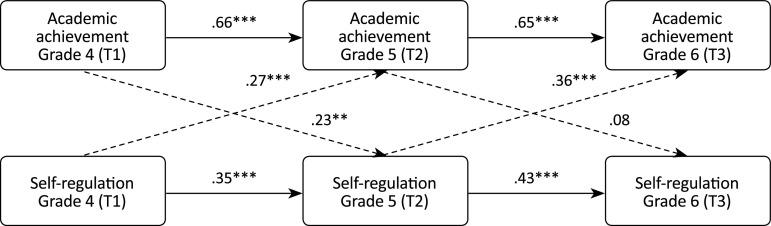
A three-wave cross-lagged panel model of Self-Regulation and Academic Achievement

Note. Standardized coefficients are presented. ***p < .001, **p < .01, *p < .05

The task of substantiating the long-term effects of conscious SR on the students’ PWB and academic performance was addressed by means of a cross-longitudinal analysis. This allowed us to assess the stability of the studied features over time, through analysis of simultaneous relationships and the overall variation of variables within each of the measurements, as well as to reveal causal relationships, showing how much the variation in the previous measurement of one feature explains the variation in the subsequent measurement of another feature. *Figures 4* and *5* present the final models, including only significant relationships.

The results confirmed the role of conscious self-regulation of primary school students as a significant predictive resource for PWB and achievement during their transition to secondary school and further education. These results have been verified in several studies on different samples, using methods for diagnostics of not only psychological but also subjective well-being.

Comparative analysis of the regression coefficients of the model presented in *Figure 4* made it possible to reveal the role of students’ PWB in overcoming difficulties of the transition period and ensuring further academic success. Not only the level of conscious SR achieved at the end of primary school, but also the high level of PWB, serve as the foundation for further SR development. Indeed, 5th grade, with its transition to radically new learning conditions, requires a higher development of regulatory competencies. At the stage of adaptation to these conditions, conscious SR becomes a key resource that contributes to the academic performance and PWB of adolescents.

Comparison of the significance and magnitude of the regression coefficients in the model presented in *Figure 5* allow us to state that the level of students’ academic achievement in 4th grade predicts the development of their self-regulation in 5th grade. However, upon transition to 6th grade, it is conscious self-regulation that predicts academic performance. If we compare these results with the dynamics of SR development, it can be noted that transition from 4th to 5th grade is associated with significant changes in the self-regulation of students’ learning activity. These changes become an effective resource for maintaining academic performance in secondary school. Our research allowed to identify and describe this effect.

## Discussion

The study tested the hypothesis that self-regulation, being the controlling meta-level of the system of universal and special regulatory resources for achieving educational goals, a) directly affects academic success, and b) mediates the influence of other individual reserves on it, especially that of psychological well-being. The results expand the scientific understanding of the age-related dynamics of conscious self-regulation and psychological well-being as psychological resources for the academic performance of young adolescents.

In particular, we have shown that during the transition from primary to secondary school, along with gradually growing well-being, there is an improvement in academic performance and self-regulation in 5th grade, and a temporary deterioration in 6th grade. Previously, it was believed that difficulties for students arise precisely in 5th grade, which probably predetermined the increased attention of teachers and parents to this period and provided timely assistance to fifth-graders.

We showed that, contrary to popular belief about the difficulties of the transition from primary to secondary school (with the threat of declining PWB), the number of students with negative PWB dynamics (12% of the sample) turned out to be significantly lower than the number of students with positive (22%) and stable PWB dynamics (67%) (Morosanova et al., 2019). This empirical result allows to conclude that the majority of schoolchildren are able to actualize their psychological resources for overcoming the difficulties of the transition period. On the one hand, fifth-graders receive significant social support from parents and teachers, and, on the other, being in a new situation, they gain new experience in self-organization of their activities and social interaction, becoming more independent and autonomous in solving educational problems. This life experience becomes the basis for a positive attitude towards oneself and contributes to the formation of high self-esteem. But the lack of real experience of independence leads to the fact that they now face the difficulties of independence in the 6th grade.

It is important to note that PWB dynamics during transitions rarely attract the attention of researchers due to their instability. Thus, students who had the highest level of PWB in 4th grade have a lower level in 5th grade, and vice versa, students who initially had the lowest PWB demonstrate its significant increase in 5th grade (Morosanova et al., 2019). The few longitudinal studies of PWB dynamics in adolescents show somewhat conflicting results. Thus, Herke and colleagues (Herke, Rathmann & Richter, 2019) found a decrease in well-being from grade 5 to grade 12; Yang and colleagues from grade 9 (Yang, Tian, Huebner & Zhu, 2019). Very few studies have proposed heterogeneous models of the students’ PWB dynamics. One of them, conducted on a sample of 687 students, covered the educational transition in middle and late adolescence in four waves, describing three subgroups of life satisfaction trajectories: stable-high, high-decreasing, and low-increasing (Pande et al., 2018). These results are supported by the study of Tikhomirova et al., who described three trajectories of life satisfaction in adolescents: high-stable, declining, and improving (Tikhomirova, Malykh, & Malykh, 2020).

Analysis of non-cognitive predictors of academic performance in groups with different PWB dynamics has demonstrated its scientific promise. As shown in our study of academic performance in the group with increasing PWB, its high level is ensured by the development of Planning and Responsibility competencies. In the stable PWB group, the determinants of academic achievement – regulatory Flexibility and Modeling – have an instrumental function and are able to provide only the already achieved level of academic success, whereas low academic performance in the group with decreasing PWB can be explained by the lack of regulatory competencies. Thus, identifying the resource role of self-regulation in relation to PWB and academic achievement allows for a fresh look at SR, taking into account the fact that resources can both accumulate and be depleted. A similar view of self-regulation as a depletable resource can be found in the works of R. Baumeister on ego-depletion (Baumeister et al., 2018).

Previous studies have shown that as children grow older, the determination of their academic performance and well-being changes. At the beginning of secondary school, each non-cognitive predictor makes a separate direct contribution to academic performance and then, by the time of transition to high school, these relationships “fold up”, with only those remaining that reliably ensure the achievement of results. This applies primarily to conscious self-regulation (Morosanova et al., 2018). Therefore, it is important to identify and evaluate mediating links. The general level of conscious self-regulation demonstrates the highest effect in the relationship between PWB and academic performance. Of the regulatory competencies, we especially note Planning and Programming, which are important abilities for the beginning of secondary school. The ability to set a goal and build specific steps to achieve it will subsequently form the basis of many positive characteristics, such as responsibility, initiative, a high level of achievement motivation, and high results in educational and professional activities. Thus, our study confirmed the hypothesis that conscious self-regulation is a universal resource for psychological well-being and academic achievement, which can affect academic performance both directly and through the relationship of special regulatory resources for the students’ psychological well-being.

A similar result was obtained by other researchers (Calmeiro, Camacho, & de Matos, 2018). These data indicate that adolescence can be an important period both for the development of self-regulation and for the actualization of its meta-resource role.

A theory’s value depends upon its predictive power. One result of our study was its predictive models that answer the question: does the level of SR development predict psychological well-being and academic performance in the long term? The analysis of the longitudinal relationship between conscious SR, PWB, and academic achievement made it possible to reveal the mechanisms of their mutual determination. The transition from primary to secondary school is the unique schooling period where we can observe the reciprocity of the relationship between PWB and SR. In the future, such reciprocity manifests itself in the relationship between SR and academic performance, SR and school engagement (see Fomina, Filippova, & Zhemerikina, 2022; Morosanova, Bondarenko, & Kondratyuk, 2021). Both models demonstrate that 5th grade is a significant stage in the development and formation of conscious self-regulation, as well as its actualization as a meta-resource for academic achievement and well-being of adolescents. Many studies have appeared in which self-regulation is considered a significant mechanism for maintaining the psychological well-being of adolescents. One of them identified the relationships between positive development and self-regulation (Gestsdottir & Lerner, 2008); it has also been shown that the skills of self-control and self-regulation in adolescents provide higher levels of subjective well-being and life satisfaction (Chang et al., 2020); another study revealed the role of self-regulation as a meta-resource preventing the formation of problem behavior in adolescence (Galton, Morrison, & Pell, 2000). The results of our study are consistent with those of other researchers. Thus, stable linear positive relationships were obtained: high academic performance is associated with a higher level of subjective well-being of students (e.g., Converse et al., 2018; Salmela-Aro & Tynkkynen, 2010). At the same time, longitudinal studies have found that the relationship between academic achievement and PWB can be reciprocal: a high level of PWB can be both a precursor and a consequence of academic achievement (Robson, Allen, & Howard, 2020). Researchers insist that practical work on the development of self-regulation (in its various manifestations) has great potential for increasing life satisfaction among adolescents (Tolan & Larsen, 2014). All this also emphasizes its role as a managing meta-resource not only for academic success, but also for solving various age-related problems.

From a theoretical point of view, our results convincingly show the meta-resource nature of SR, since without it, neither PWB nor academic achievement have a reliable psychological basis that ensures their stable development. In addition, the analysis of the trajectories of SR, PWB, and academic achievement is of high practical importance for the early identification of “problem” areas in adolescents’ development and helps to effectively allocate resources in the designing of psychological interventions (Herke, Rathmann, & Richter, 2019).

## Conclusion

The study provides new evidence for the resource role of conscious self-regulation in predicting the levels of PWB and academic success of students during their transition from primary to secondary school.

The data analysis establishes heterochronous changes in the SR general level and academic achievement of students during the transition period. The significant rise of these indicators, recorded upon transition to 5th grade, is replaced by a decline upon transition to 6th grade. The psychological well-being of schoolchildren in this period is, on average, characterized by systematic positive dynamics, but it is advisable to consider groups of students with increasing, stable, and decreasing PWB levels.

We have shown that conscious self-regulation is a universal resource for both academic performance and psychological well-being of students. The study also revealed some special resources for academic achievement and students’ well-being, depending on the trajectory of changes in their PWB. Improved well-being is determined by the regulatory competencies of Planning and Results Evaluation, while its stability is determined by Planning, Modeling, Flexibility, and Responsibility.

The data analysis revealed and described the mediator effects of conscious SR on the relationship between psychological well-being and academic success of young adolescents. The most pronounced mediator effect is typical for the regulatory-cognitive competences of Planning and Programming, the regulatory-personal feature of Responsibility, as well as the general level of conscious self-regulation.

The study established significant long-term effects of the positive impact of self-regulation on the psychological well-being and academic performance of students. Prognostic effects are demonstrated not only for the general SR level, but also for certain regulatory components.

The data on various trajectories of PWB and SR dynamics make it possible to set new priorities for the age development of young adolescents, directing the efforts of teachers, psychologists, and parents towards developing the students’ self-regulation in 5th grade and maintaining their well-being in 4th grade.

## Limitations

The present study did not analyze gender differences in students’ psychological well-being, nor did it assess school satisfaction among the adolescents. Investigating these variables in future studies will take into account their contextual impact.
